# Tau protein- induced sequestration of the eukaryotic ribosome: Implications in neurodegenerative disease

**DOI:** 10.1038/s41598-020-61777-7

**Published:** 2020-03-23

**Authors:** Senjuti Banerjee, Sehnaz Ferdosh, Amar Nath Ghosh, Chandana Barat

**Affiliations:** 1 Department of Biotechnology, St. Xavier’s College, Park Street, Kolkata, 700016 West Bengal India; 2 National Institute of Cholera and Enteric Diseases P-33, C.I.T. Road, Scheme XM, Beleghata, India

**Keywords:** Biochemistry, Molecular biology

## Abstract

The human tau is a microtubule-associated intrinsically unstructured protein that forms intraneuronal cytotoxic deposits in neurodegenerative diseases, like tauopathies. Recent studies indicate that in Alzheimer’s disease, ribosomal dysfunction might be a crucial event in the disease pathology. Our earlier studies had demonstrated that amorphous protein aggregation in the presence of ribosome can lead to sequestration of the ribosomal components. The present study aims at determining the effect of incubation of the full-length tau protein (Ht40) and its microtubule binding 4-repeat domain (K18) on the eukaryotic ribosome. Our *in vitro* studies show that incubation of Ht40 and the K18 tau variants with isolated non-translating yeast ribosome can induce a loss of ribosome physical integrity resulting in formation of tau-rRNA-ribosomal protein aggregates. Incubation with the tau protein variants also led to a disappearance of the peak indicating the ribosome profile of the HeLa cell lysate and suppression of translation in the human *in vitro* translation system. The incubation of tau protein with the ribosomal RNA leads to the formation of tau-rRNA aggregates. The effect of K18 on the yeast ribosome can be mitigated in the presence of cellular polyanions like heparin and tRNA, thereby indicating the electrostatic nature of the aggregation process.

## Introduction

Protein aggregate formation and their intracellular accumulation are associated with a wide range of neurodegenerative proteinopathies. The Alzheimer’s disease (AD) is a neurodegenerative disease characterised by formation of neurofibrillary tangles (NFTs) that are composed of hyperphosphorylated tau proteins^1^. In addition to Alzheimer’s disease, abnormal aggregation of the tau protein has been linked to the pathogenesis of more than 20 other neurodegenerative disorders, collectively termed as tauopathies^1^. Although a link between pathological tau aggregation and cognitive impairment has been established, the mechanism by which tau aggregation causes neuronal dysfunction is unclear.

Several lines of evidence suggest that the ribosome is a cellular interacting partner of the tau protein. Earlier immunohistochemical studies using tau specific antibody have demonstrated that, there is an association of the tau protein with both, the microtubule and the ribosome in the neuronal cells^2^. More recent studies of the human tau interactome have also revealed a robust association of the tau protein with the ribonucleoproteome and these studies have indicated at the preferential association of the full-length tau protein Ht40 to the 80S ribosome^3^. Although aggregation of the tau protein is the primary event in AD, it has also been demonstrated that the impairment of cellular translation and ribosome dysfunction, due to tau-ribosome interaction, is an early event in the disease. Such disruption of protein synthesis machinery might contribute towards the neuronal loss which characterises the disease^4^. This is also in agreement with earlier studies which noted that a decline in protein synthesis and ribosome function is initiated during mild cognitive impairment (MCI) that develops into AD^5,6^. Although a profound loss in the ribosomal complexes in the affected regions of the brain have been shown to occur in parallel to the progression of the disease^7^, the reasons underlying the disappearance of the ribosomal peaks is not completely understood.

The tau protein is a microtubule associated protein that promotes the assembly and stabilization of the microtubule structure^8^. Hence, tau mediated neurodegeneration might arise due to either the loss of physiological function or the gain of toxic function. Tau aggregation would abolish its microtubule stabilizing function and hence, impair neuronal transport. However, the absence of significant neuronal abnormalities in tau knock-out mice^9^ have suggested that the loss of tau function may be less critical and the gain of toxic function in the process of tau aggregate formation might be the key factor in neurodegeneration. Several recent evidences also suggest that sequestration of cellular interacting partners (proteins or RNA) by protein aggregates could contribute to pathogenesis in a wide spectrum of neurodegenerative diseases^10^. The fact, that the intrinsically unstructured tau protein is capable of interacting with polyanionic cofactors like heparin^11^ and cellular RNAs^12^, raises the possibility that the tau protein is also capable of interacting with highly charged macromolecular complexes such as the ribosome. Our earlier studies had shown that the partial unfolding or amorphous aggregation of lysozyme and Bovine Carbonic Anhydrase II (BCAII), in the presence of empty non-translating prokaryotic or eukaryotic ribosome, could induce aggregation of ribosomal components^13^. Hence, based on these observations, the present study aims at determining the effect of incubation of the tau protein on the physical integrity of the eukaryotic 80S ribosome.

Our *in vitro* experiments demonstrate that the incubation of tau variants (K18 and Ht40) with the eukaryotic yeast 80S ribosome leads to a dose dependent and progressive loss of ribosomal peak, that is indicative of the loss of ribosome population. A disappearance of the ribosome peak and decline in *in vitro* translation ability of the HeLa cell lysate containing the human ribosome is also observed upon incubation with the tau variants, indicating a similar loss of physical integrity of the human ribosome. The incubation of the tau variants with isolated yeast ribosomal RNA leads to the formation of large tau-rRNA containing aggregates. Studies have also been performed on the effect of the cellular polyanions like heparin and tRNA (that are known tau protein interactors) on the outcome of Ht40- and K18- ribosome co-aggregation.

## Results

### Structure and electrostatics of human tau protein and yeast ribosome

**Figure 1:**
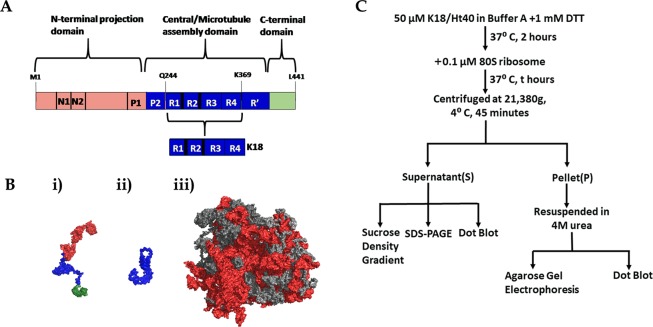
Structure of human full-length Tau and the yeast ribosome. (**A**) *A schematic representation of the Tau protein*: Schematic diagram of the human full-length tau protein Ht40 and its microtubule binding repeat domain K18, highlighting the functional and electrostatic differences between its domains. The “N terminal domain” has a calculated net negative charge of −14.9 (red), the “Central domain” has a calculated net positive charge of +20.1 (blue) and the “C-terminal domain” has a nominal negative charge of −3.1 (green). The R1, R2, R3 and R4 sequences constitute the K18 protein (blue) which has calculated a net charge of +10. All charges are calculated at pH 7.5 using PROTEIN CALCULATOR v3.4. (**B**) *Surface representation of the Ht40 and K18 model structures obtained using i-TASSER and Saccharomyces Cerevisiae ribosome (*PDB ID: 3Z22 and 3O58) [*using PYMOL 2008 (De Lano Scientific, Palo Alto, CA, USA, available at:* http://www.pymol.org]. The models of Ht40 (Radius of gyration: 6.5 ± 0.3 nm; Mylonas *et.al*., Biochemistry, 2008) and K18 (Radius of gyration: 3.8 ± 0.3 nm; Mylonas *et.al*.,Biochemistry, 2008) displayed here were chosen based on the C-score. (i) Ht40 model: The N terminal domain has been represented in red, the positively charged central domain is represented in blue and the C terminal domain is represented in green (The colour coding is same as in A); (ii) Model of K18; (iii) Solvent exposed surface of the yeast 80S ribosome subunit (PDB ID: 3Z22 and 3O58). The rRNA is represented in red and the ribosomal proteins are represented in gray. (**C**) An outline of the experimental procedures followed in this study is depicted in the form of a flowchart.

Electrostatics have been shown to play a major role in determining the interactions of tau with its cellular partners^14^. Intrinsically unstructured proteins are also known to engage in non-native electrostatic interactions with their cognate cellular partners^15^. The electrostatic features of the tau protein variants used in our study and the ribosomes, which therefore might play a major role in the tau-ribosome interaction, is highlighted in Fig. 1. Figure 1A shows the full-length isoform of the tau protein Ht40 and its subdomain K18, whose effect on the ribosome forms the subject of this study. The longest isoform of human Tau, 2N4R or Ht40, is divided into 3 functional domains: (a) the projection domain consisting of the N-terminal acidic region and proline rich domains, (b) the central microtubule assembly domain containing the pseudo-repeat regions and (c) the C terminal domain^11^. The full length tau protein has been colour coded based on the predominant charges of its corresponding domains and highlights that the Ht40 is a dipole with oppositely charged domains. The wide variability in the pI of the domains results in a considerable difference in their net charge at physiological pH^16^. This renders the N-terminal domain acidic (red), the central domain basic (blue) and the C-terminal domain neutral (green) as shown in Fig. 1A. The positively charged K18 tau variant, that corresponds to the central microtubule binding repeat domains (R1, R2, R3 and R4), is represented in blue. The conformation of the two tau variants, Ht40 and K18 as predicted by the I-TASSER server^17–19^, is shown in Fig. 1Bi,Bii and the colour coding of the domains based on the net charge is the same as in Fig. 1A. The ribosomal RNA (rRNA) present in the solvent exposed surfaces of the yeast 80S ribosome has been highlighted in red (Fig. 1Biii). Electrostatic calculations have indeed revealed that the ribosome surface is predominantly negatively charged^20^ and the rRNA has a major contribution in negative electrostatic potential of the ribosome^21^. A flowchart denoting the basic procedure of the *in vitro* experiments performed in the study discussed below is shown in Fig. 1C and detailed in the “Materials and methods section”.

### Effect of Tau on eukaryotic ribosomes

**Figure 2:**
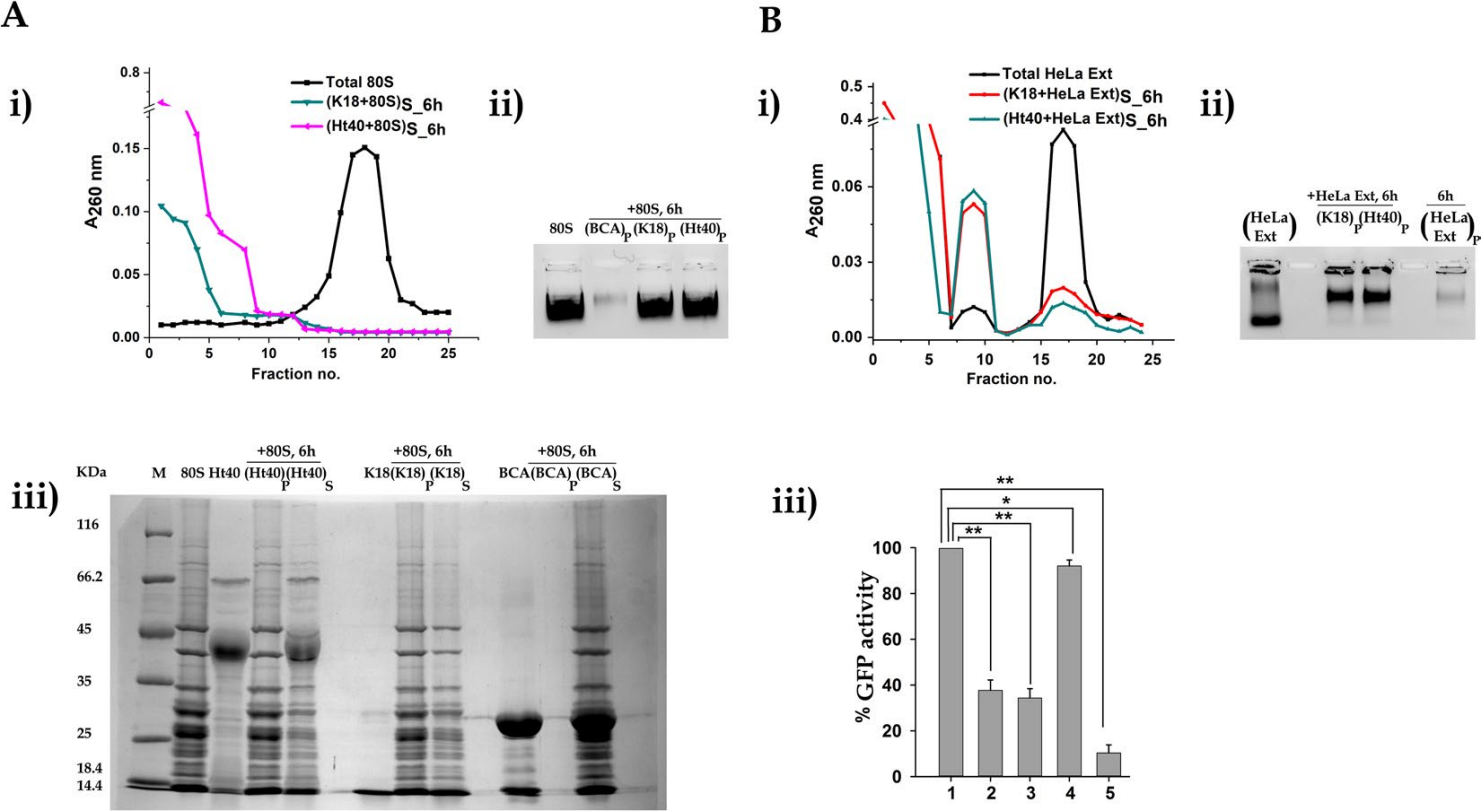
Effect of tau on eukaryotic ribosome. Effect of tau variants K18 or Ht40 on eukaryotic ribosome was studied using the procedure outlined schematically in Figure 1C. Briefly, 0.1 µM purified yeast 80S ribosome was incubated with 50 µM tau variant for 6 hours at 37 °C in Buffer A (25 mM Tris-HCl pH 7.5, 50 mM NaCl, 5 mM MgCl_2_) and the resultant reaction mixture was centrifuged. The supernatant fractions were analysed using sucrose density gradient centrifugation (SDGC). The sedimentation profile of the ribosome in the supernatant fraction was obtained by plotting A_260 nm_ against the fraction number (“S” in the subscript indicates the supernatant fraction and the number indicates the time of incubation in hours). Disappearance of the ribosomal peak was observed when the 80S ribosome was incubated with K18 or Ht40 (Ai). The pellet fractions (indicated by “P” in the subscript along with appropriate time of incubation denoted in hours) were resuspended in 4 M Urea containing Buffer A and analysed for the presence of Aii) ribosomal RNA (using 0.8% agarose gel electrophoresis) and for Aiii) ribosomal protein (using 12% SDS PAGE: materials and methods). Control experiments were performed where yeast 80S ribosome was similarly incubated in presence of native BCAII (BCA), centrifuged and analysed using electrophoretic methods (Aii, Aiii). (**A**) *Effect of tau variants on purified yeast 80S ribosome*. (**i)** Sedimentation profile of the supernatant: (1) Total 80S ribosome (■), (2) (K18 + 80S)_S _6h_ (), (3) (Ht40 + 80S)_S_6h_ (). (**ii**) Agarose gel electrophoretic analysis of pellet for the presence of ribosomal RNA; lanes from left to right contain: (1) Total 80S, (2) (BCA + 80S)_P_6h_, (3) (K18 + 80S)_P_6h_, (4) (Ht40 + 80S)_P_6h_ (**iii**) SDS PAGE analysis of the pellet and supernatant for the presence of ribosomal proteins; lanes from left to right contain: (1) Molecular weight marker, (2) Total 80S, (3) Ht40 total protein, (4) (Ht40 + 80S)_P_6h,_ (5) (Ht40 + 80S)_S_6h_, (6) Blank, (7) K18 total protein, (8) (K18 + 80S)_P_6h_, (9) (K18 + 80S)_S_6h,_ (10) Blank, (11) BCAII total protein, (12) (nBCAII+80S)_P_6h_, (13) (nBCAII+80S)_S_6h_, (14) Blank. (**B**). *Effect of tau variants on human 80S ribosomes present in HeLa cell lysate*. The HeLa cell lysate or extract (ext) used in our experiments is a component of the human IVT kit. (**i**) Equivalent A_260 nm_ units (to 0.1 µM of yeast 80S ribosome) of HeLa cell lysate was incubated in the presence of 50 µM K18 or Ht40 for 6 hours, under reducing conditions, at 37 °C and the resultant reaction mixture was analysed as stated in materials and methods. (**i)** Sedimentation profile of HeLa extract (ext) in the supernatant fraction; Total HeLa ext (■), (K18 + HeLa ext)_S_6h_ (), (Ht40+HeLa ext)_S_6h_ (). (**ii**) Agarose gel electrophoretic analysis of pellet for the presence of RNA**;** Lanes from left to right contain: (1) Total HeLa ext, (2) Blank, (3) (K18 + HeLa ext)_P_6h_, (4) (Ht40+HeLa ext)_P_6h_, (5) Blank, (6) (HeLa ext)_P_6h_ (**iii**) *In vitro transcription-translation assay using human coupled IVT kit and tau variants*. 50 µM K18 or Ht40 or nBCAII was added to the reaction mixture of human *in vitro* translation system and incubated at 30 °C for 6 hours (as prescribed by the manufacturer). Bar graphs show the percentage GFP activity in the presence and absence of tau protein variants. The GFP fluorescence activity observed in the reaction mix containing the plasmid and neither of the tau variants is considered as 100%. (1) + GFP plasmid, (2) 50 µM K18 + GFP plasmid, (3) 50 µM Ht40 + GFP plasmid, (4) 50 µM nBCAII + GFP plasmid, (5) - GFP plasmid. The experiment has been repeated thrice and the data are presented as means ± SEM; *P < 0.05 or **P < 0.001 in one –way ANOVA (N = 3).

As stated in the introduction section, the effect of incubation of the tau protein on the eukaryotic 80S ribosome is the principal objective of the present study. As stated earlier, the flowchart in Fig. 1C outlines the basic procedures that have been followed, in which 50 µM of reduced tau variants (Ht40 and K18) were incubated with 0.1 µM of purified yeast 80S ribosome under physiological conditions, (37 °C and pH 7.5: materials and methods). The conditions of the experiments with respect to concentrations of the tau variants and the salt concentrations used, were optimum for tau aggregation^22^. However, no additional cellular polyanions that are necessary for inducing tau aggregation were present during the incubation. The centrifugal speed used in our study differs from the speed used earlier in polyA RNA induced tau aggregation studies^23^, for separation of monomeric tau from tau-RNA aggregates. At this speed (1,00,000 g), the ribosome or rRNA alone is pelleted irrespective of the presence of the tau proteins. Hence, at the centrifugal speed used in our studies (21,380 g), the larger aggregates would form the pellet (P) while the supernatant (S) might be constituted of the residual ribosomes and smaller aggregates. In our initial experiments the tau variants were incubated with the ribosome for 6 hours. The supernatant fractions obtained after Ht40-80S and K18-80S incubation were analysed by sucrose density gradient centrifugation (SDGC) and the A_260 nm_ profile obtained was compared to the profile of equivalent amount of untreated yeast 80S ribosome. As shown in Fig. 2Ai, a significant reduction in the ribosomal peak and a simultaneous appearance of peaks of ribonucleoprotein particles with lower sedimentation coefficients was observed when the ribosome was incubated with the tau protein variants. The disappearance of the ribosome peak might thereby indicate a global disassembly of the 80S ribosome and aggregation of its components that is also suggested by the experiments discussed in the next section.

The constituents of the pellet and supernatant fractions were also analysed using electrophoretic methods. Agarose gel electrophoretic analysis of the pellet fractions (Fig. 2Aii) showed that the aggregates in the pellet [that were formed in the presence of the tau variants (Ht40 and K18)] constituted of a major proportion of the ribosomal RNA. The method used for agarose gel electrophoresis is as described in the “Materials and methods” section. This method enables us to visualise the total rRNA present in the ribosome as a single consolidated band, as has been used in previous studies on tau-rRNA interactions^24^. In the presence of suitable controls, this method has also been used in our earlier studies as a semi-quantitative technique for following protein-ribosome aggregation process^13^. Similarly, analysis of the protein components of the pellet and the supernatant fractions by denaturing SDS-PAGE was also done which showed that upon incubation of the 80S ribosome with the tau variants K18 and Ht40 (for 6 hours), the presence of ribosomal proteins was observed, both in the supernatant as well as in the pellet (Fig. 2Aiii**)**. Control experiments were performed in which the yeast ribosome (0.1 μM) was incubated in the presence of the native bovine carbonic anhydrase II (nBCAII) protein (50 μM), under similar conditions, and centrifugation was performed as stated earlier. As shown in Fig. 2Aii, insignificant amount of ribosomal RNA was observed in the pellet, thereby indicating that the large rRNA containing aggregates were formed, specifically when the ribosome is incubated with the tau proteins. Also, upon incubation of 80S ribosome with native BCAII under similar conditions, all the ribosomal proteins were retained in the supernatant fraction (Fig. 2Aiii), thus reaffirming that aggregation of ribosomal components is subject to the specific presence of the tau protein variants. Additional experiments performed, showed that the 80S ribosome was retained in the supernatant when incubated alone or with DTT under similar conditions as stated above (Fig. S1A). Further control experiments were performed to verify whether a contaminant that co-purified during recombinant expression and purification of the tau protein might have led to ribosome aggregation. As described in the “materials and methods” section, the *E. coli* cells expressing the tau protein variants were lysed using the “direct boiling method” and the cellular extract obtained (A_280 nm_ units equivalent to 50 µM of K18) was incubated with 0.1 µM yeast 80S ribosome for 6 hours and centrifuged. As shown in Fig. S1B, no ribosomal proteins were present in the pellet fraction at the speed of centrifugation used in our studies thus confirming that that the aggregation of ribosomal components was specific to the presence of tau protein variants and not caused by any contaminant contributed by the cell extract.

Surprisingly, it was noted from the SDS PAGE profile that both K18 and Ht40 appeared to be present in the supernatant fraction (Fig. 2Aiii). This might be due to the limited period (6 hrs) for which the tau protein had been incubated with the ribosome during which all the tau protein had not been included in the large aggregates, which constitute the pellet. Hence, the time period of tau-ribosome incubation was extended to 24 hours, in order for the process to reach completion, and centrifuged. The presence of K18 and Ht40 in the supernatant and the pellet was analysed by dot-blot analysis using K18 and Ht40 specific monoclonal antibodies (materials and methods). The total rRNA content of the supernatant was also estimated using A_260 nm_ values. As shown in Fig. S1Ci,ii, both K18 and Ht40 are present in the pellet as well as in the supernatant and a significant proportion of the total rRNA is also retained in the supernatant (Fig. S1Ciii). Electron microscopy of an aliquot of the supernatant obtained by centrifugation after 24 hours of K18–80S incubation also showed the presence of aggregates (Fig. S1Civ), lending support to the fact that at the centrifugal speed used in our experiments, a proportion of ribosome-tau aggregates is still retained in the supernatant fraction. Thus, it is possible that the supernatant contains small co-aggregates of tau and ribosomal components. Taken together, these studies do however establish that the Tau protein is the initiator of ribosome disassembly and aggregation of ribosomal components. Further experiments were performed to study the dependence of tau-ribosome aggregation on the concentration of the tau protein present. As shown in Fig. S1Di,ii,S1Ei,ii, the dose dependent reduction of the 80S ribosome peak and increase in appearance of the ribosomal RNA in the insoluble fraction occurs in presence of increasing concentration of both variants of the tau protein (K18 and Ht40) and the ribosome. Therefore, it could be implied that upon increasing the concentration of the tau protein (condition that promotes tau aggregation), the extent of tau induced ribosome aggregation is also increased.

It should be noted that the tau protein is a major protein of the human neuronal cells. Hence, whether this protein has a similar effect on the human ribosome as well, needed to be addressed. In order to study the effect of the tau protein on human ribosomes, the tau variants were incubated with the cell lysate obtained from the HeLa cell line (Materials and Methods). This cell lysate represents a heterogeneous system that includes the translational machinery as one of its significant components and the A_260 nm_ profile obtained by sucrose density centrifugation can reflect the total ribosomal population present in the lysate. Hence, in our subsequent experiment, the HeLa cell lysate containing 0.1 μM equivalent A_260 nm_ units was incubated with 50 µM of tau proteins K18 or Ht40 for 6 hours, centrifuged and the A_260 nm_ profile (obtained by SDGC) of the supernatant was compared to that of the untreated lysate. A significant alteration of the A_260 nm_ profile (Fig. 2Bi) and an accumulation of sub-ribonucleoprotein particles was observed upon incubation of the lysate with the tau protein. The pellet obtained after centrifugation of the aggregation mix contained a significant amount of the RNA (a substantial proportion of which is rRNA^25^) (Fig. 2Bii) and of proteins present in the cell extract (Fig. S1Fi). In presence of the tau variants, a concomitant reduction of cellular RNA is observed in the supernatant fraction as analysed using agarose gel electrophoresis (Fig. S1Fii). However, the additional peak in the SDGC profile (Fig. 2Bi) and the shift in the mobilities of the RNA (Fig. 2Bii) that is observed only in the presence of the tau proteins need to be further investigated. The effect of the tau proteins on the translational ability of the HeLa cell lysate was also analysed. In this study, GFP was used as a reporter gene (Materials and methods). As shown in Fig. 2Biii, the fluorescence activity of the GFP reporter protein, measured 6 hours after the initiation of translation, was significantly reduced in the presence of the (50 μM) tau protein variants. No significant change in GFP activity was observed in presence of equivalent amounts of the native BCAII protein. Earlier studies had also indicated that the tau protein might possess the ability to modulate the translation process^4,26^. Hence the translational suppression in presence of the tau protein variants implies that the loss of physical integrity of the ribosomal population (as observed by change in A_260 nm_ profile in Fig. 2Bi) is also reflected in the loss of functional integrity of the ribosome.

Although, further *in vitro* and *in vivo* experiments need to be performed with purified human ribosomes and disease affected neurons respectively, our studies imply that tau-ribosome interaction could underlie the change in ribosome profile observed in diseased neurons. Further, based on the observed similarity in the effect of tau variants on both yeast and human ribosomes and the fact that yeast is established as a model system for studies of neurodegenerative diseases^27^, all subsequent experiments in this study were performed with purified yeast 80S ribosome.

### Time course of tau induced ribosome aggregation

**Figure 3:**
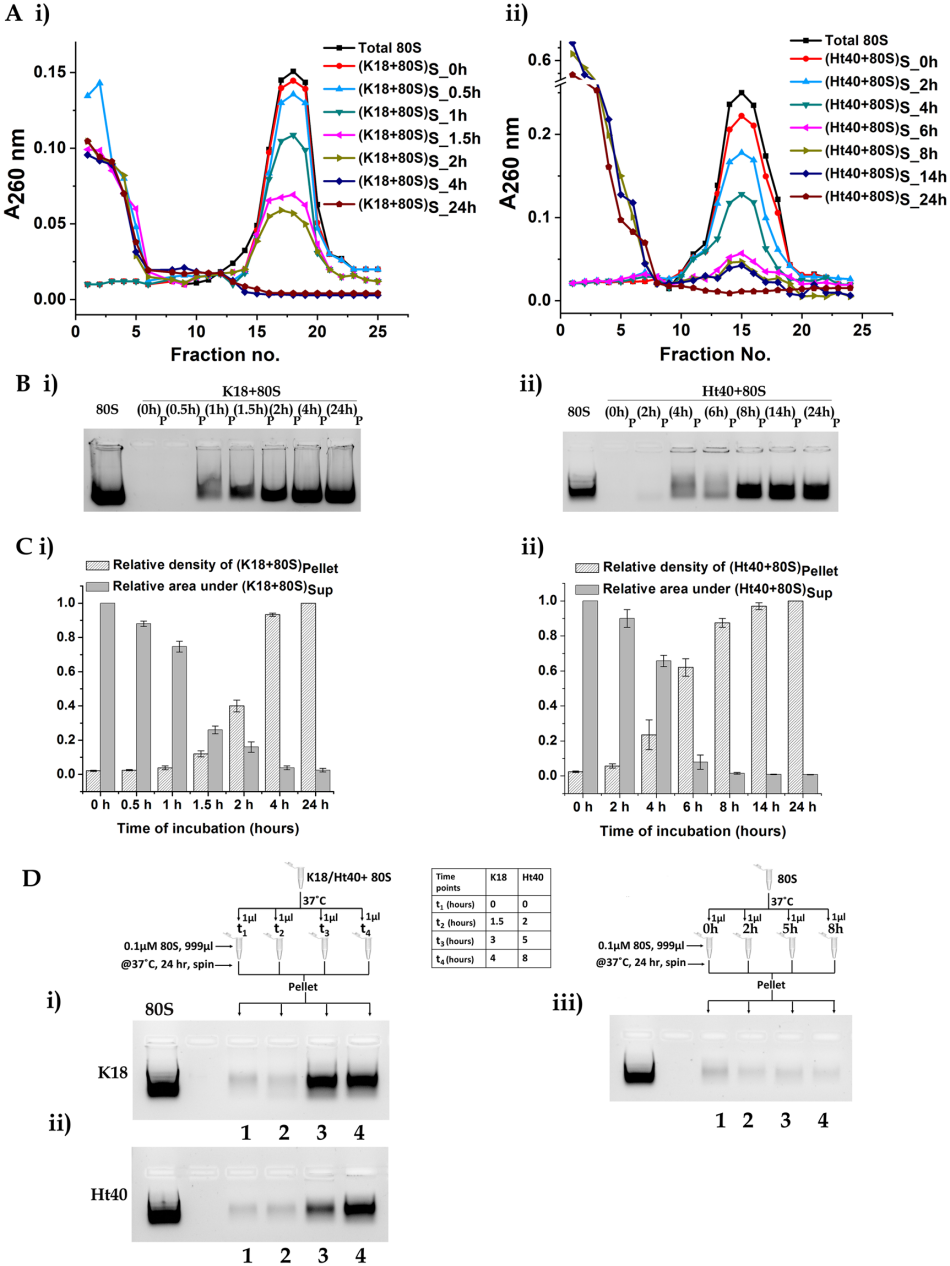
Time dependence of tau induced yeast 80S ribosome aggregation, The yeast 80S ribosome (0.1 µM) was incubated with the tau variants (50 µM) for different time intervals, centrifuged and the supernatant and pellet fractions were analysed as stated in materials and methods. (**A**) (i) *Sedimentation profile of the supernatant obtained at different time intervals for K18-80S aggregation*: Total 80S ribosome (■), (K18 + 80S) _S_0h_ (), (K18 + 80S)_S_0.5h_ (), (K18 + 80S)_S_1h_ (), (K18 + 80S)_S_1.5h_ (), (K18 + 80S)_S_2h_ (), (K18 + 80S)_S_4h_ () and (K18 + 80S)_S_24h_ (). (**ii)** *Sedimentation profile of the supernatant obtained at different time intervals for Ht40-80S aggregation*: Total 80S ribosome (■), (Ht40 + 80S)_S_0h_ (), (Ht40 + 80S)_S_2h_ (), (Ht40 + 80S)_S_4h_ (), (Ht40 + 80S)_S_6h_ (), (Ht40 + 80S)_S_8h_ (), (Ht40 + 80S)_S_14h_ () and (Ht40 + 80S)_S_24h_ (). (**B**) (**i**) *Agarose gel electrophoretic analysis of pellet obtained at different time intervals for K18-80S aggregation for the presence of ribosomal RNA*: Lanes from left to right contain; (1) Total 80S ribosome, (2) (0 h)_P_, (3) (0.5 h)_P_, (4) (1 h)_P_, (5) (1.5 h)_P_, (6) (2 h)_P_, (7) (4 h)_P_, (8) (24 h)_P_ (**ii**) *Agarose gel electrophoretic analysis pellet obtained at different time intervals for Ht40-80S aggregation for the presence of ribosomal RNA*: Lanes from left to right contain; (1) Total 80S ribosome, (2) (0 h)_P_, (3) (2 h)_P_, (4) (4 h)_P_, (5) (6 h)_P_, (6) (8 h)_P_, (7) (14 h)_P_, (8) (24 h)_P_. (**C**) (**i**) *Area under 80S peak in supernatant and densitometry of rRNA in pellet for K18-80S aggregation:* Bar graphs representing the relative intensity obtained after densitometric scanning of the rRNA bands in the pellet fraction on agarose gel electrophoresis and relative area under the peak of 80S ribosome in the soluble fraction at different time intervals of K18-80S aggregation. [(K18 + 80S)_P_24h_ assumed as 1 for densitometry calculations whereas the area under the 80S peak at 0 h has been assumed as 1 for area under the 80S peak calculations]. (**ii)** *Area under 80S peak in supernatant and densitometry of rRNA in pellet for Ht40-80S aggregation:* Bar graphs representing the relative intensity obtained after densitometric scanning of the rRNA bands in the pellet fraction on agarose gel electrophoresis and relative area under the peak of 80S ribosome (profile in SDGC)/ in the soluble fraction at different time intervals of Ht40-80S aggregation. [(Ht40 + 80S)_P_24h_ assumed as 1 for densitometry calculations whereas the area under the 80S peak at 0 h has been assumed as 1 for area under the 80S peak calculations]. (**D**) *Ability of tau ribosome aggregates to induce aggregation of untreated ribosome:*Seeding experiments with tau-ribosome aggregates were performed as stated in materials and methods. Briefly, 0.1 µM 80S ribosome was incubated with 50 µM K18 or Ht40 at 37 °C. 1 µl aliquots were withdrawn from the reaction mixtures at specified time points and added to 999 µl of fresh 80S ribosome, which was then incubated for 24 hours, centrifuged and the pellet analysed using agarose gel electrophoresis. A schematic representation of the seeding experiments is included in the Figure. Aliquots drawn at respective time points are indicated as (t)_al_. *Agarose gel electrophoresis of rRNA in the pellet when 0.1 µM 80S was incubated with aliquots of Tau-80S aggregation mix withdrawn at different time points:* (**i)** For K18 + 80S induced aggregation, lanes from left to right contain: (1) Total 80S, (2) Blank and 80S ribosome incubated with aliquots withdrawn at (3) 0 h, (4) 1.5 h, (5) 3 h, (6) 4 h (**ii)** For Ht40 + 80S induced aggregation lanes from left to right contain: (1) Total 80S, (2) Blank and 80S ribosome incubated with aliquots withdrawn at (3) 0 h, (4) 2 h, (5) 5 h, (6) 8 h (**iii)** Control experiments show that 80S ribosome which was not previously incubated with K18 or Ht40 was incapable of inducing ribosome aggregation. Lanes 3-6 (numbered as 1–4) are 80S ribosome incubated with aliquots of untreated ribosome withdrawn at 0 h, 2 h, 5 h and 8 h respectively.

In order to explore the time course followed by the tau-ribosome aggregation process, the tau-variants (50 µM) were incubated with the ribosome (0.1 µM) for different time periods and centrifuged. The supernatant and pellet fractions were analysed by SDGC and gel electrophoresis. The sucrose density gradient profiles of the supernatants in Fig. 3Ai,Aii show that for increasing periods of K18–80S and Ht40–80S incubation, a progressive disappearance of the ribosomal peak with the appearance of small ribonucleoprotein particles is observed. Agarose gel electrophoresis of the corresponding pellets also showed an increase in intensity of the rRNA band, upon increasing the time of tau-ribosome incubation for both K18 (Fig. 3Bi) and Ht40 (Fig. 3Bii). The supernatant and pellet fractions obtained at selected time points after initiation of tau-ribosome incubation, were also analysed using denaturing SDS-PAGE in order to determine the presence of ribosomal proteins. A time dependant appearance of the overall ribosomal proteins in the pellet with their reduction in the supernatant is observed (Fig. S2Ai,ii). These observations indicate that at a high tau:80S ratio of 500:1, a process of global disassembly of the 80S ribosome might be initiated with the formation small and large aggregates constituted of tau and ribosomal components. The tau- ribosome aggregation process, visualized by low resolution electron microscopy (materials and methods), also shows the formation of large heterogeneous aggregates at 24 hours of tau-ribosome incubation (Fig. S2B,C) while no such aggregation was apparent when the tau variants were incubated alone in the absence of ribosome (Fig. S2D). Our preliminary studies, as stated above, shows that the tau protein, which initiates the process of disassembly and aggregation of the ribosomal components, is present both in the supernatant and pellet. However, further studies need to be performed in order to analyse the (a) partitioning of the tau, rRNA and ribosomal proteins into large and small aggregates, (b) aggregation status of the tau proteins and (c) its association with the ribosomal components.

At each time point of incubation, the area under the 80S peak in the supernatant and the intensity of the rRNA band in the pellet provides a semi-quantitative measure for following the tau-ribosome aggregation process. Hence, the experiments in Fig. 3Ai,Aii,3Bi and 3Bii were performed multiple times and the relative intensities of the rRNA in the pellet and area under the 80S peak in the SDGC profile of the supernatant are represented in the form of a bar diagram (Fig. 3Ci,Cii). In this representation, the intensity of the rRNA pellet band obtained upon 24 hours incubation of the tau variants with the ribosome was considered as 100%. This was done on basis of the observation that a substantial proportion of the rRNA is still retained in the supernatant (as sub-ribonucleoprotein particles) even after 24 hours of tau-ribosome incubation (Figs. S1Biii, 3A,ii). As shown in the Fig. 3Ci, a sharp rise in rRNA in the pellet fraction was observed with a concomitant decrease of the ribosomal peak in the supernatant between 1.5 to 4 hours of K18–80S incubation. Ht40 induced 80S aggregation, however, showed a drastic increase between 4 to 8 hours (Fig. 3Cii). These experiments were indicative of a cooperative nature of the tau-ribosome aggregation process and were further investigated by performing the seeding experiments as stated below.

In the seeding experiments aliquots from the reaction mixture containing the ribosome and tau variants (K18 or Ht40) were withdrawn at different time points (as indicated in the Fig. 3D) and added to a thousand-fold excess of fresh (untreated) 0.1 µM 80S ribosome (materials and methods). These mixtures were incubated for 24 hours at 37 °C, centrifuged and the insoluble pellet fractions were analysed using agarose gel electrophoresis. A schematic representation of the experiments performed is included in the Figure (Fig. 3D). It was observed that, the aliquot, when withdrawn after the 3hrs of K18- ribosome incubation and 5 hrs of Ht40-ribosome incubation, was sufficient to induce significant aggregation of the new 80S ribosome that had not previously been incubated with the tau protein (Fig. 3Di,Dii respectively). Appropriate control experiments were performed which showed that the aliquots drawn from 0.1 µM 80S incubated under similar conditions in the absence of any tau protein variant for the above mentioned time points (0 h, 2 h, 5 h and 8 h), could not induce the aggregation of fresh 80S ribosome (Fig. 3Diii). This study implicates a self-perpetuating nature of the ribosome aggregation process once it is initiated by the tau- ribosome interaction. Whether the aggregated ribosomes or a component formed due to tau-induced ribosome disassembly and co-aggregation has provided this potential to self-perpetuate their own aggregation needs to be further studied.

### Yeast ribosomal RNA as an inducer for tau aggregation

Since the ribosome is a complex multicomponent ribonucleoprotein particle, the question arises as to whether a particular component is primarily responsible for the tau-ribosome aggregation process. As stated in the introduction section, the natural tendency of the tau protein to interact with cellular polyanions presents the anionic surface of the ribosome, largely constituted of rRNA (Fig. 1), as a potential candidate for tau-ribosome aggregation. Previous studies have shown that tau aggregate formation can also be induced upon incubation with cytoplasmic RNA, a major proportion of which is rRNA^25^. Several studies have also shown that RNA can influence the aggregation of prion proteins^28,29^. Interestingly, cytoplasmic RNA has been detected in pathological lesions associated with diverse neurodegenerative diseases^30^.

**Figure 4:**
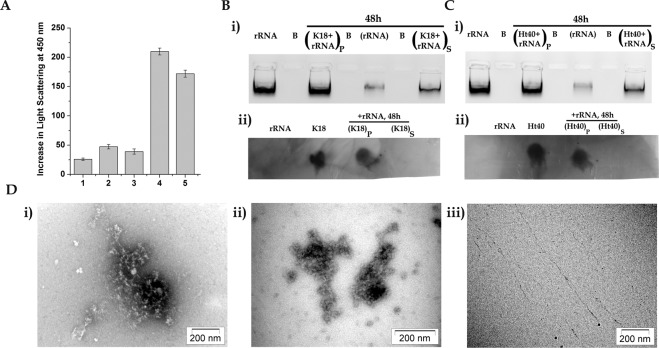
Yeast ribosomal RNA can lead to the formation of rRNA-tau coaggregates. 50 µM K18 or Ht40 was incubated in the absence and presence of 1 µM 80SrRNA for 48 hours at 37 °C as described in “Materials and Methods”. The aggregation was monitored by change in light scattering intensity and substantial increase in scattering was observed after 48 hours of tau-rRNA incubation. The reaction mixture was centrifuged, the supernatant and pellet fractions were analysed using dot blot and agarose gel electrophoresis. (**A**). *Light Scattering studies:* Bar graphs representing change in light scattering intensity at 450 nm after 48 hours of incubating K18 or Ht40 (50 µM) in the presence of 80SrRNA (1 µM) (Materials and Methods): (1) rRNA alone, (2) K18 alone (3) Ht40 alone, (4) K18 + 80SrRNA, (5) Ht40 + 80SrRNA. (**B**). Analysis of supernatant and pellet obtained from K18-80SrRNA aggregation i) *Agarose gel electrophoresis analysis:* Lanes from left to right indicate; (1) Total 80SrRNA, (2) Blank, (3) (K18 + rRNA)_P_48h,_ (4) Blank, (5) (rRNA)_P_48h_, (6) Blank, (7) (K18 + rRNA)_S_48h_ ii) *Dot blot analysis using anti-K18 monoclonal IgG antibody:* Dots from left to right indicate; Total 80SrRNA, Total K18, (K18 + rRNA)_P_48h_, (K18 + rRNA)_S_48h_. The full-length dot-blot image is shown in Fig. S4Bi. (**C**). Analysis of supernatant and pellet obtained from Ht40-80SrRNA aggregation i) *Agarose gel electrophoresis analysis*: Lanes from left to right indicate; (1) Total 80SrRNA, (2) Blank, (3) (Ht40+rRNA)_P_48h,_ (4) Blank, (5) (rRNA)_P_48h_, (6) Blank, (7) (Ht40+rRNA)_S_48h_ (ii) *Dot blot analysis using anti-Ht40 monoclonal antibody:* Dots from left to right indicate; Total 80SrRNA, Total Ht40, (Ht40+rRNA)_P_48h_, (Ht40+rRNA)_S_48h_. The full-length dot-blot image is shown in Fig. S4Bii. (**D**). *Transmission electron microscopic analysis of tau-rRNA aggregates:* Micrographs were prepared from samples withdrawn at 48 hours from the initiation of incubation of 1 µM 80SrRNA with or without 50 μM of K18 or Ht40 (materials and methods): Micrographs of (i) (K18 + 80SrRNA)_48h_, (ii) (Ht40 + 80SrRNA)_48h_, (iii) (80SrRNA)_48h_.

Hence, subsequent studies were performed on the effect of incubation of tau variants with rRNA isolated from the yeast 80S ribosome. In this study 1 µM of extracted 80S rRNA (as described in materials and methods) was incubated with 50 µM of K18 or Ht40 for 48 hours at 37 °C and the change in light scattering intensity at 450 nm was measured. As shown in Fig. 4A, a significant increase in light scattering intensity was observed when K18 and Ht40 were incubated with 80S rRNA, in comparison to when the tau variants and the rRNA were incubated alone.

In order to further analyse the aggregates formed, the reaction mixture was centrifuged and the pellet and supernatant fractions were analysed using agarose gel electrophoresis and dot blot analysis (materials and methods). As shown in Fig. 4Bi, for K18-rRNA incubation, the rRNA indeed forms a major component of the pellet fraction, although a portion of it is still retained in the supernatant fraction. Dot blot analysis of the soluble and insoluble fractions obtained upon K18-rRNA incubation (materials and methods) showed that almost the entire amount of K18 protein is present in the pellet fraction, in presence of rRNA (Fig. 4Bii). Similar observations were made in the agarose gel (Fig. 4Ci) and dot blot analysis (Fig. 4Cii) of the supernatant and pellet obtained upon Ht40-rRNA incubation. Whether the rRNA:tau ratio of 1:50, unlike the 80S ribosome:tau ratio of 1:500 and the extended period of tau-rRNA incubation (48 h) are responsible for the presence of tau entirely in the pellet needs to be further analysed. Electron microscopy of the aggregates obtained upon incubating both K18 and Ht40 with rRNA at 37 °C for 48 hours showed the formation of intertwined fibrils (Fig. 4Di,Dii respectively), having appearance similar to that observed upon incubation with ribosome. These experiments, were performed with the total 80SrRNA and the contribution of specific rRNA (28S and 18S) requires further analysis. In the control experiment, the incubation of the rRNA alone under similar conditions showed no apparent aggregate formation (Fig. 4Diii).

In this context, it should be noted that recent studies show that RNA can influence aggregation of disease-associated proteins^28,31^. A recent study with recombinant prion protein (rPrP) has showed that depending upon the protein: RNA stoichiometric ratio, the RNA is capable of inhibiting or stimulating protein aggregation. At a high protein: RNA ratio, rPrP aggregation^28^ like tau aggregation in our study, is stimulated, also leading to its co-aggregation with the RNA. Our experiments also collectively indicate that the rRNA component of the ribosome could play a significant role in the ribosome-tau aggregation phenomenon observed here.

### Effect of cellular anions on tau-ribosome aggregation

Earlier studies have demonstrated the importance of electrostatic interactions in determining the interaction of tau with its cellular partners and that the behaviour of the tau protein can be modulated in presence of cellular polyanions^14^. Hence, further studies were performed to investigate the possibility of inhibition of tau initiated ribosome aggregation by electrostatic shielding agents in the form of competitor polyanions like heparin and tRNA. In these experiments the tau variants were incubated with the ribosome in presence of increasing concentrations (0x, 1x, 5x, 10x; x = 0.1 µM) of tRNA or heparin, centrifuged and the aggregation process was followed by agarose gel electrophoresis of rRNA present in the insoluble tau-ribosome aggregates. The highest concentrations of tRNA and heparin used in these experiments were 10-fold higher than that of the 80S ribosome, as was used in our earlier studies^13^. Also, as reported in literature, the cellular stoichiometric ratio of tRNA with respect to the ribosome in yeast is approximately 10-fold^32^.

**Figure 5:**
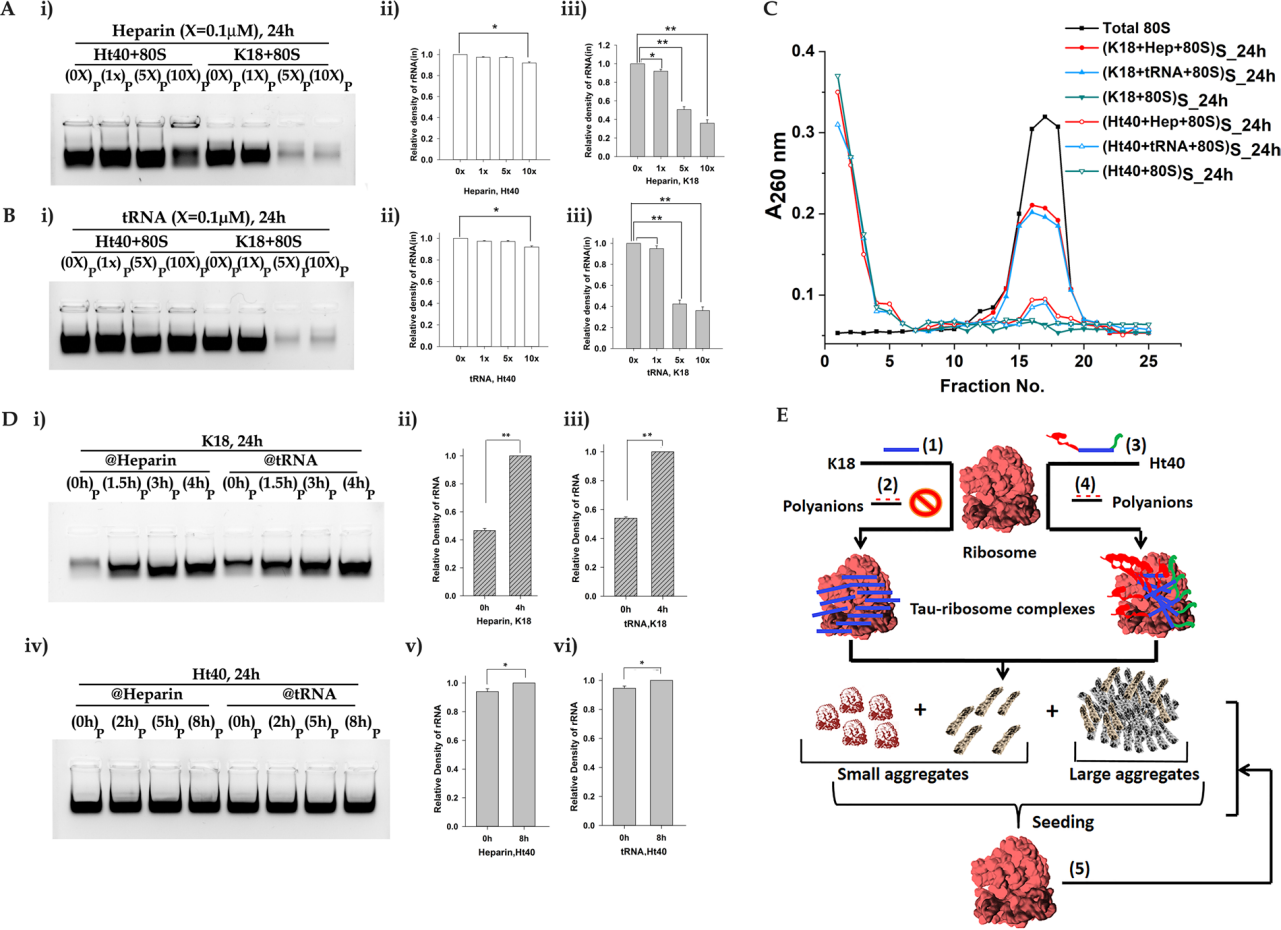
Effect of polyanions on Tau induced 80S aggregation. 0.1 µM of 80S ribosome was incubated with 50 µM K18 or Ht40 in presence of increasing concentrations (0x, 1x, 5x and 10×; × = 0.1 µM) of heparin (hep) or yeast phenylalanine tRNA for 24 hours (Materials and Methods). The reaction mixture was centrifuged, the pellet and supernatant were analysed as described before. (**A,B**) *Agarose gel electrophoretic analysis of rRNA in the pellet.* (**A**). Effect of heparin: i) Lanes from left to right contain: (1) (Ht40 + 0x hep+80S)_P_, (2) (Ht40 + 1x hep+80S)_P_, (3) (Ht40 + 5x hep+80S)_P_, (4) (Ht40 + 10x hep+80S)_P_, (5) (K18 + 0x hep+80S)_P_, (6) (K18 + 1x hep+80S)_P_, (7) (K18 + 5x hep+80S)_P_, (8) (K18 + 10x hep+80S)_P_; (ii) Bar graphs depicting the densitometric analysis of rRNA band intensities show no suppression of aggregation in presence of heparin for Ht40-80S aggregation. (Ht40 + 0x heparin+80S)_P_24h_ assumed as 1 for calculations; (iii) Bar graphs depicting the densitometric analysis of rRNA band intensities show suppression of K18-80S aggregation in presence of heparin; (K18 + 0x heparin+80S)_P_24h_ assumed as 1 for calculations. The experiments were repeated thrice and the data are presented as means ± SEM; *P < 0.05 or **P < 0.001 in one –way ANOVA (N = 3). (**B**). Effect of tRNA**:** (i) Lanes from left to right contain: (1) (Ht40 + 0x tRNA+80S)_P_, (2) (Ht40 + 1x tRNA+80S)_P_, (3) (Ht40 + 5x tRNA+80S)_P_, (4) (Ht40 + 10x tRNA+80S)_P_, (5) (K18 + 0x tRNA+80S)_P_, (6) (K18 + 1x tRNA+80S)_P_, (7) (K18 + 5x tRNA+80S)_P_, (8) (K18 + 10x tRNA +80S)_P_; Bar graphs were plotted as in (**A**)and depicts the densitometric analysis of rRNA band intensities. Bar graphs (**Bii)** [(Ht40 + 0x tRNA+80S)_P_ assumed as 1] and (**iii)** [(K18 + 0x tRNA+80S)_P_ assumed as 1] shows selective suppression of aggregation K18-80S aggregation but no suppression of Ht40-80S aggregation in presence of tRNA. The experiments were repeated thrice and the data are presented as means ± SEM; *P < 0.05 or **P < 0.001 in one –way ANOVA (N = 3). (**C**). *Sedimentation profile of supernatant fraction*: 80S was incubated with K18 or Ht40 in presence of 10×(x = 0.1 µM) heparin or tRNA and the sedimentation profile was observed for; (1) Total 80S (■), (2) (K18 + hep+80S)_S_24h_ (), (3) (K18 + tRNA+80S)_S_24h_ (), (4) (K18 + 80S)_S_24h_(), (5) (Ht40+hep+80S)_S_24h_ (), (6) (Ht40+tRNA+80S)_S_24h_ (), (7) (Ht40 + 80S)_S_24h_ (). Retention of 80S peak was observed with heparin and tRNA selectively in case of K18-80S aggregation. (**D**). *Effect of delayed addition of heparin and tRNA*: 0.1 µM 80S was incubated with 50 µM of Tau variants (K18 or Ht40) and 1 µM (10×) heparin or tRNA was added to the reaction mixture at different time points from the initiation of incubation (Materials and Methods). The reaction mixture was further incubated till 24 hours, centrifuged and rRNA in the pellet was analysed using agarose gel electrophoresis. (i) K18-80S aggregation for delayed addition of heparin and tRNA: Lanes from left to right contain pellets for K18-80S aggregation when heparin was added at: (1) 0 h, (2) 1.5 h, (3) 3 h, (4) 4 h and when tRNA was added at (5) 0 h, (6) 1.5 h, (7) 3 h, (8) 4 h. Bar graphs (ii) and (iii) depicting the densitometric analysis of relative intensities of rRNA bands (Intensity of pellet band for 4 h assumed as 1) in the pellets for **Di** show that delayed addition of heparin and tRNA shows no effective of suppression in case of K18-80S aggregation. (iv) Ht40-80S aggregation for delayed addition of heparin and tRNA: Lanes from left to right contain pellets for Ht40-80S aggregation when heparin was added at: (1) 0 h, (2) 2 h, (3) 5 h, (4) 8 h and when tRNA was added at (5) 0 h, (6) 2 h, (7) 5 h, (8) 8 h. Bar graphs (v) and (vi) shows the densitometric analysis of relative intensities of rRNA bands (Intensity of pellet band for 8 h assumed as 1) that revealed the lack of any effect of the time of heparin or tRNA addition on the Ht40-80S aggregation process. The experiments were repeated thrice and the data are presented as means ± SEM; *P < 0.05 or **P < 0.001 in one–way ANOVA (N = 3). (**E)** *A model showing the fate of the ribosome upon encountering the Ht40 and K18 Tau variants*. The tau protein variants Ht40 and K18 associate with the RNA rich polyanionic ribosome surface. The possible aggregation of tau on the exposed rRNA initiates the process of co-aggregation of tau and ribosomal components leading to a time dependent formation of small and large RNA protein co-aggregates. The highly positively charged K18 variants associates with the ribosome surface predominantly via electrostatic interactions (1), that is effectively inhibited in presence of cellular polyanions like tRNA and heparin (2). The full-length tau variant Ht40 having an additional large unstructured N-terminal domain associates with the ribosome via electrostatic and additional interactions (3) which is not effectively shielded in the presence of added polyanions (4). The tau (K18 and Ht40) ribosome aggregates are capable of seeding aggregation of fresh untreated ribosomes (5). This cartoon representation has been created using Adobe Photoshop CS 8.0.

As shown in Fig. 5, increasing concentrations of heparin could significantly inhibit K18-ribosome aggregation, that is reflected as a reduced formation of insoluble rRNA containing aggregates (Fig. 5Ai–iii). No such inhibition of Ht40 induced ribosome aggregation was observed (Fig. 5Ai and represented in the bar diagram Fig. 5Aii,Aiii). Selective inhibition of K18–80S aggregation was also observed in presence of yeast tRNA, under the conditions used in our experiment (Fig. 5Bi and represented in the bar diagram Fig. 5Bii,Biii). The sucrose density gradient profile of the soluble fraction also showed a significant retention of the 80S peak in the presence of the highest concentration of polyanions for K18 induced ribosome aggregation. No such protective effect of the polyanions was observed on the ribosomal peak for Ht40 induced 80S aggregation (Fig. 5C). Control experiments were performed to verify whether the heparin and tRNA concentrations used in our experiments could themselves lead to tau or ribosome aggregation. When the ribosome was also incubated with these polyanions for 24 hours in absence of the Tau protein variants, no significant appearance of rRNA was observed in the insoluble pellet (Fig. S3A), confirming that the polyanions themselves do not induce ribosome aggregation. Also, in control experiments, K18 or Ht40 were incubated with different concentrations of heparin and tRNA (used in our above stated experiments), for 24 hours, centrifuged and the insoluble pellet was analysed on SDS-PAGE (Fig. S3Bi,ii). It was observed that, the polyanions even at their highest concentrations (1 µM) could not themselves induce aggregation of the ribosome or the tau variants.

We also analysed whether the time of addition of these polyanions could affect tau induced ribosome aggregation. As shown in Fig. 5D(i–iii), presence of these polyanions from the initiation (t = 0 h of ribosome-tau incubation) of the aggregation process was necessary for effective suppression of ribosome aggregation induced by K18. Also, as expected from the above observations (Fig. 5B), the time of inducer addition showed no such effect in case of Ht40 induced 80S aggregation (Fig. 5Div–vi).

From the selective effect of the polyanions, it can be concluded that under our experimental conditions, electrostatics plays a major role in determining the outcome of K18–80S aggregation process. Further reasons that might underlie the difference between K18 and Ht40–80S aggregation are discussed below and is depicted in the model (Fig. 5E).

## Discussion

As stated in the introduction section, a significant change in the 80S ribosome profile occurs in the neurons of individuals afflicted with Alzheimer’s disease and a gradual decrease in the neuronal ribosome population is observed with the progression of the disease^6,7^. Our *in vitro* studies show that when full length tau protein (Ht40) and the K18 tau variants were incubated *in vitro* with isolated non-translating yeast ribosome under conditions that are conducive to tau aggregation (but in absence of polyanionic inducer), the aggregation of ribosomal components was induced. The tau protein variants had a similar effect on the ribosome profile of the HeLa cell lysate and suppression of translation by the human *in vitro* translation system was also observed in presence of the tau protein variants. The concentration and time dependence of tau mediated ribosome aggregation and the ineffectiveness of BCAII protein to induce 80S ribosome aggregation are indicative of a ribosome aggregation process that is subject to the specific presence of the tau proteins. Further *in vivo* experiments need to be performed with the disease affected neurons to confirm our observations made *in vitro*. Our studies imply that the tau proteins alone might mediate the change in ribosomal profiles observed in neurons of the Alzheimer’s affected regions of human brain^7^, which might be a vital determinant of tau aggregation toxicity.

The ability of tau-ribosome co-aggregates to seed the aggregation of ribosomes is another surprising phenomenon observed in this study. This study implies that the tau-ribosome aggregates formed can successfully propagate the aggregation of new ribosomes without requiring the presence of freshly added tau proteins. It might be noted that earlier studies have shown that when the K18 variant of the tau protein, rPrP and the p53 proteins were incubated with RNA, protein-RNA aggregates formed could act as seeds to nucleate *de novo* protein aggregation^23,28,31^. Since the eukaryotic ribosome is a large ribonucleoprotein particle, a large amount of tau- rRNA and tau-ribosomal protein aggregates are formed during the tau-ribosome aggregation process. As RNA molecules have been observed to contribute to the seeding behaviour of RNA-protein aggregates (as stated earlier), it might be suggested that the ribosome, majorly consisting of rRNA molecules, might rely on the rRNA component for this seeding behaviour. In addition, the large number of ribosomal proteins with intrinsically disordered regions can also play a significant role. Hence, further studies need to be performed to identify the component formed due to tau-ribosome co-aggregation that possesses the potential to self-perpetuate the ribosome aggregation process.

Another question that arises is what forms the basis for the tau-ribosome interaction that eventually culminates into the co-aggregation process reported in this study. Several recent studies have highlighted that the electrostatic interactions between the unfolded nascent polypeptide chain and the ribosome have diverse effects on translation rate^33^ and co-translational protein folding^34^. Interestingly, an unrelated study has also implicated that the ribosome surface properties may limit the mobility of positively charged variants of recombinant GFP in *E. coli*^21^. The formation of aggregates upon incubation of the purified ribosomes with the positively charged GFP variant indicates at the tendency of a positively charged protein to induce ribosome aggregation. Our earlier studies have also suggested that electrostatics plays a role during co-aggregation of lysozyme (which has a net positive charge at physiological pH) with the ribosome^13^. However, the contribution of net charge on a protein towards its ability to bring about ribosome aggregation needs further investigation. Recent studies have demonstrated that the disease associated intrinsically disordered proteins are highly charged and often rely on non-native electrostatic interactions for associating even with their appropriate cellular binding partners^35,36^. Such a tendency of engaging in promiscuous interactions might also lead to cytotoxicity. Indeed, it has been demonstrated that electrostatic interaction of the tau protein with anionic lipid membranes can induce tau aggregation and that the resultant membrane permeabilization may serve as a pathway by which tau protein aggregates exert their toxicity^37,38^. Earlier studies had demonstrated that cellular RNA (a major portion of which is rRNA) can induce tau fibrillization^27^ and RNA modulated prion aggregation is also widely reported in literature^28,29^.

Our studies also show that the incubation of the tau variants with the rRNA isolated from the 80S ribosome could lead to the formation of tau-rRNA aggregates. The ability of the rRNA to induce rRNA-Tau aggregation implicated that the rRNA component of the ribosome plays a dominant role in this process, although, the participation of ribosomal proteins containing regions of intrinsic disorder^39^, in the tau-ribosome aggregation process, also requires to be further investigated. However, in light of present studies and taking into account the relative size and concentration of the eukaryotic ribosome and the tau variants, it is possible that the anionic surface of the ribosome, that is predominantly composed of rRNA, could present itself as a potential surface on which multiple tau molecules can associate (Fig. 5E). Such associations could induce tau aggregation by either screening of the intermolecular electrostatic repulsions between tau proteins or by increasing the local tau concentration. Since the rRNA acts as an architectural scaffold for the ribosome, such tau-rRNA interactions and aggregation of tau on the ribosome surface could lead to disruption of the ribosome structure. Such tau-targeted structural destabilization of the translational machinery could lead to cytotoxicity, although this model (Fig. 5E) needs to be further verified. Intriguingly, recent studies have also indicated that ribosomes are especially susceptible to the protein aggregates that are formed either due to abnormal protein stoichiometry during aneuploidy or due to protein aggregation in aged brains^40,41^. Our recent and earlier^13^ studies suggest that the ribosome when placed in the vicinity of protein aggregate formation have a tendency to co-aggregate, thereby providing a plausible explanation to the predominance of ribosomal proteins in the aggregates observed in these studies^40,41^. The inhibition of tau-ribosome electrostatic interactions in the presence of added polyanions, like heparin and tRNA, indeed could inhibit K18 induced tau aggregation. However, the lack of inhibition of Ht40 mediated ribosome aggregation in presence of the polyanions might be either due to the difference in the net positive charge of K18 and Ht40 at physiological pH (Fig. 1) or because of additional interactions between the large unstructured N-terminus of the full-length tau protein and the ribosome. It should be noted here that, the specific interactions between the chemically unfolded protein and the ribosomal RNA, that forms the basis of the ability of the ribosome to act as a protein folding modulator, has been widely reported in literature (^42^ and the references therein). Whether such tau-ribosome interactions play a role in the observed co-aggregation of the tau proteins with the ribosome needs to be investigated.

The question also arises about whether such tau-ribosome interactions would become more probable under pathological conditions and whether such interactions are a cause or consequence of the AD pathology. Such a probability of aberrant non-translating ribosome-tau interactions might increase in AD, in which the mutated tau proteins (P301L tau mutant)^3^ and the differentially truncated forms of tau lose their affinity for microtubules, thus altering their subcellular localization and increasing the cytosolic tau concentration^43–45^. Studies on the abilities of other tau isoforms to initiate the tau-ribosome aggregation process also need to be performed. Preliminary studies conducted with the 3-repeat domain containing tau variant (K19) show that similar to K18 variant, K19 is able to aggregate the ribosome (Fig. S3C), although further experiments are necessary to characterize the K19-tau mediated ribosome aggregation process. Earlier studies also suggest that there exists a correlation between the glycosylation and phosphorylation status of the tau protein and AD. However, it should be noted that our studies have been conducted with tau proteins expressed in bacterial cells and hence lack any such post-translational modification. Further investigations are also necessary to determine the effects of such disease associated changes in post-translational modifications on the ability of tau protein to induce ribosome aggregation^46^. The age dependent decline in both protein translation^47^ and cellular chaperone^48^ levels might further increase the possibility of promiscuous tau-ribosome interactions. The interference in biological functions of the tau protein as discussed above and its increased aggregation propensity are the early events in AD. Although, the tau-ribosome aggregation is likely to be a consequence rather than a cause for AD, our present studies indicate that the disruption of cellular translation machinery might provide important insights into the processes that lead to Alzheimer’s disease pathology.

## Materials and Methods

### Materials

Bovine Carbonic Anhydrase II (BCAII), NaCl, MgCl_2_, DTT, Tris-base, SP sepharose were purchased from Sigma Aldrich. Tau 4RD (K18), Tau 3RD (K19) and Ht40 clones were obtained as kind gifts from Dr.Jayant B. Udgaonkar’s Laboratory (NCBS). Polyallomer ultracentrifuge tubes were purchased from Beckman Coulter. Nitrocellulose filter was purchased from Bio-Rad, PVDF membrane was purchased from Millipore, mouse monoclonal anti-human Tau 4RD antibody was purchased from EMD Millipore (primary antibody for K18) [Anti-Tau (4-repeat isoform RD4) Antibody, clone 1E1/A6; Catalogue no.: 05–804], mouse monoclonal anti-mouse D8 antibody (primary antibody for Ht40) [Tau Antibody (D8), Catalogue no.: sc-166060] and the goat-anti-mouse HRP conjugated antibody (Secondary antibody) were purchased from Santa-Cruz Biotechnology. The western chemiluminescence HRP substrate was purchased from Millipore (Immobilon). Yeast phenylalanine tRNA was purchased from Sigma-Aldrich. All other chemicals were local products of analytical grade. Experimental data analysis was performed using OriginPro8 (Origin Corp., Northampton, MA, USA), QuantityOne Bio-Rad and SIGMA-PLOT 14 (Systat Software, Inc., San Jose, CA, USA) software. PYMOL 2008 (De Lano Scientific, Palo Alto, CA, USA) was used to display the Protein Data Bank files. Net charge calculations of human full-length tau (Ht40) and K18 were performed using PROTEIN CALCULATOR v3.4.

### Buffers

Buffers used in this study were: Buffer A for aggregation reactions: 25 mM Tris-HCl pH 7.5, 50 mM NaCl, 5 mM MgCl_2_ (with minor modifications from Ramachandran *et.al*., 22); Buffer B for cell lysis: 50 mM PIPES NaOH pH 6.9,20 mM NaCl,1 mM EDTA,5 mM DTT, 1 mM PMSF^22^; Buffer C for protein purification^22^: 50 mM PIPES NaOH pH 6.9,20 mM NaCl; Buffer D for protein purification^22^: 50 mM PIPES NaOH, pH 6.9 500 mM NaCl; Buffer E for final buffer exchange and protein storage^22^: 25 mM Tris-HCl pH 7.5.

### Purification of Tau K18, K19, Ht40 and yeast 80S ribosome

Yeast *Saccharomyces cerevisiae* ribosome (80S) was purified according to Chakraborty *et al*.^49^. Yeast 80S rRNA was extracted as mentioned in Piir *et al*.^50^. Tau K18 and Ht40 were purified using a two-step purification method: direct-boiling method^51,52^ and cation-exchange purification method^22^. The *E. coli* BL21(DE3) cells were transformed with Tau k18, k19, ht40 plasmids and cells were harvested for large scale purification by centrifugation. The cell pellets were resuspended and boiled in Buffer B for 20 minutes with gentle agitation after every 5 minutes. The boiled cell suspension was immediately chilled on ice for 15 minutes before centrifuging it at 4 °C and 16,000 g for 20 minutes. The supernatant was retained and incubated with SP sepharose for cation exchange chromatography and washed with increasing concentrations of NaCl (obtained my mixing Buffer C and Buffer D)^22^. Fractions containing pure protein were pooled together, concentrated and finally buffer exchanged into Buffer E and frozen at −80 °C until further use. The protein concentration was determined as mentioned in Ramachandran *et al*.^22^.

### Tau-ribosome aggregation

The tau aggregation procedure and buffer composition were followed from Ramachandran *et.al*.^22^ with minor modifications. 50 µM Tau K18 or Ht40 was reduced in presence of 1 mM DTT for 2 hours at 37 °C in Buffer A, at the end of which 0.1 µM 80S or 1 µM 80S rRNA or 1 µM heparin or 1 µM tRNA or A_260 nm_ units equivalent to 0.1 µM 80S (7.9 A _260 nm_ units) HeLa cell lysate was added and incubated for 6 hours at 37 °C or as mentioned. For aggregation reactions with increasing concentrations of tau, 10 µM, 25 µM and 50 µM of K18 and Ht40 was used. In control experiments 50 µM native BCAII (nBCAII) was incubated with 0.1 µM 80S for 6 hours at 37 °C. 80S (0.1 µM) was also incubated in the presence and absence of 1 mM DTT for 6 hours in buffer A and centrifuged. Aggregation reactions were centrifuged at 21,380 g for 45 minutes at 4 °C and the pellet and supernatant fractions separated. It should be noted that the centrifugal speed used in earlier studies on polyA RNA induced tau aggregation^23^ to separate monomeric tau and tau-RNA aggregates was 1,00,000 g which could not be used for our studies, since at this centrifugal speed the ribosome or rRNA alone is pelleted irrespective of the presence of the tau proteins. Hence, at the centrifugal speed of 21,380 g^13^, the larger aggregates formed the pellet (P), while the supernatant (S) constituted of the residual ribosomes or smaller aggregates. The constituents of the pellet fractions were analysed using 12% SDS-PAGE or 0.8% agarose gel electrophoresis and the supernatant fractions by sucrose density gradient centrifugation or SDS-PAGE after TCA precipitation. The SDS-PAGE were stained using Coomassie Brilliant Blue at 37 °C and the agarose gels were stained using ethidium bromide.

### Gel electrophoretic analysis of insoluble aggregates

Aggregation samples were centrifuged at 21,380 g for 45 minutes at 4 °C. The pellets and supernatant were analysed for their total rRNA content in a non-denaturing 0.8% agarose gel, using a procedure used earlier to study interaction of tau with cellular RNA (with minor modifications)^24^. Briefly, the pellets had to be treated with 4 M urea and incubated for 20 minutes at room temperature (a treatment that was necessary to enable the RNA in the large aggregates to enter the gel), before loading on a 0.8% agarose gel for electrophoresis. The electrophoresis was performed in 1xTAE at 65 V for 10 min before visualizing under ultraviolet light using the GelDoc imaging system (MEGA BIO-PRINT 1100/20 M). In this experiment, the total RNA runs as a single band, in which the intensities of the rRNA bands were compared by densitometric analysis (QuantityOne Bio-Rad). This procedure had also been used in our earlier studies to follow the lysozyme ribosome co-aggregation process^13^. The total ribosome or rRNA used in the experiment was treated similarly and analysed. For the analysis of the protein constituents, the protein in the supernatant was TCA precipitated and the pellets were resuspended in Laemmli buffer containing 4 M urea and boiled before loading onto a 12% SDS-PAGE.

### Sucrose density gradient centrifugation

Aggregation reactions were centrifuged at 21,380 g for 45 minutes at 4 °C and the supernatant fraction was layered on a 17–25% sucrose gradient. The gradient was centrifuged at 1,98,000 g (MLS-50 rotor, Beckman Coulter) for 2.5 hours at 4 °C and 200 µl fraction volumes were collected from top to bottom and absorbance at 260 nm was measured for analysing the 80S ribosome profile.

### Dot blot analysis for tau-ribosome aggregation

50 µM K18 or Ht40 was reduced in 1 mM DTT at 37 °C for 2 hours after which 0.1 µM 80S was added to it. The resultant reaction mixture was incubated at 37 °C for 24 hours and centrifuged at 21,380 g, 4 °C for 45 minutes. The supernatant and the resuspended (in 1 M Urea) pellet fractions were dotted on PVDF membrane. The membrane was blocked with 5% skimmed milk for 1.5 hours and then washed three times (3 minutes intervals) with 1x PBST and incubated with specific primary antibody (Anti-Tau 4RD antibody monoclonal IgG; 1:250 dilution for K18 and D8-anti tau monoclonal IgG; 1:1000 dilution for Ht40) at 4 °C for overnight. The membrane was then washed eight times (15 minutes intervals) with 1x PBST and then incubated with secondary antibody (goat anti-mouse IgG HRP conjugated; 1:10,000 dilution) for 1.5 hours at room temperature. Then the membrane was washed with 1x PBST for eight times and incubated with chemiluminiscent HRP substrate and the signal was recorded using photographic plates.

### Human *in vitro* transcription-translation assay

The *in vitro* translation assay was done using 1-Step Human Coupled IVT Kit – DNA; 88881, ThermoFisher Scientific. K18 and Ht40 were added to the prescribed reaction mix to a final concentration of 50 µM. The positive control set contained no tau proteins and the negative control set did not contain the GFP (reporter gene) plasmid. All the reaction sets were incubated till 6 hours at 30 °C (as prescribed) and GFP (reporter gene) fluorescence was monitored at ex/em: 482/512 nm. The experiment was repeated three times.

### Electron microscopy

50 µM K18 or Ht40 was reduced at 37 °C for 2 hours after which 0.1 µM 80S was added. The resultant reaction mixture was incubated for 2 and 24 hours at 37 °C. For studies with rRNA, 1 µM 80S rRNA was incubated for 48 hours with 50 µM K18 or Ht40 reduced in 1 mM DTT as described above. Imaging of aggregation in the samples was done by using a transmission electron microscope (FEI Tecnai12BioTwin) with an acceleration voltage of 120 kV. Aliquots (5 µl) containing the aggregation mixture were placed on the copper grid coated with carbon film (300 meshes) and one drop of 2% uranyl acetate was placed on the grid. The excess water was removed carefully with filter paper and the grid was left to dry in air.

### Time course study of aggregation

50 µM K18 or Ht40 was reduced at 37 °C for 2 hours after which 0.1 µM 80S was added. The resultant reaction mix was centrifuged at 21,380 g for 45 minutes at 4 °C after 0 h, 0.5 h, 1 h, 1.5 h, 2 h, 4 h and 24 h of incubation for K18 and 0 h, 2 h, 4 h, 6 h, 8 h, 14 h and 24 h of incubation at 37 °C. The supernatant fractions were analysed using a 17–25% sucrose density gradient (as described below) and the pellets obtained were analysed using 0.8% agarose gel (as described below). The supernatant and pellet fractions obtained at 0.5 h, 2 h, 6 h and 24 h were also analysed using 12% SDS-PAGE as described above*.*

### Seeding assay for tau induced 80S aggregation

50 µM K18 was reduced in Buffer A for 2 hours at 37 °C, after which 0.1 µM 80S was added to it. 1 µl aliquots were drawn from this reaction mixture after 0, 1.5, 3, 4 hours of incubation (for K18 induced 80S aggregation) and 0, 2, 5, 8 hours of incubation (for Ht40 induced aggregation) added to 999 µl of fresh 0.1 µM 80S in Buffer A. This new reaction mixture was then incubated for 24 hours at 37 °C and then centrifuged at 21,380 g at 4 °C for 45 minutes. The insoluble fraction was resuspended in 4 M urea containing Buffer A and analysed using a 0.8% agarose gel electrophoresis.

### Dot-blot analysis for Tau-rRNA aggregation

50 µM K18 or Ht40 was reduced in 1 mM DTT at 37 °C for 2 hours after which 1 µM 80S was added to it. The resultant reaction mixture was incubated at 37 °C for 48 hours and centrifuged at 21,380 g, 4 °C for 45 minutes. The most commonly used Tau aggregation inducer for *in vitro* studies is heparin. In the presence of heparin at an appropriate stoichiometry the K18 aggregates much faster (24 hours)^22^ as compared to Ht40 (48–144 hours)^53^. Hence, the reaction was allowed to proceed till 48 hours in order for it to reach saturation. The supernatant and the resuspended (in 1 M Urea for 30 minutes at 37 °C) pellet fractions were dotted on PVDF membrane and the dot blot analysis was carried on as described above.

### Light scattering study

50 µM of K18 or Ht40 was reduced in Buffer A with 1 mM DTT for 2 hours at 37 °C after which 1 µM of LiCl extracted 80SrRNA was added to the reaction mixture (t = 0 h) and further incubated for 48 hours at 37 °C. The light scattering of the solutions was measured at t = 0 h and t = 48 h and the increase was plotted in the form of bar diagrams. For control, the rRNA alone, K18 alone and Ht40 alone reaction sets were similarly incubated and the increase in their light scattering was measured at 48 hours. Before measuring the light scattering intensity, all the solutions were pipetted three times. The intensity was measured at excitation: 450 nm and emission: 450 nm in Hitachi F-2700 spectrofluoremeter.

### Delayed heparin/tRNA addition assay for tau induced 80S aggregation

50 µM K18 was reduced in Buffer A for 2 hours at 37 °C in presence of 1 mM DTT, after which 0.1 µM 80S was added to it. 1 µM heparin or tRNA was added after 0, 1.5, 3 and 4 hours of addition of the ribosome in case of K18 and after 0, 2, 5 and 8 hours of addition of the ribosome in case of Ht40. The resultant reaction mixture was further incubated for 24 hours at 37 °C and centrifuged at 21,380 g at 4 °C for 45 minutes. The insoluble fraction was resuspended in 4 M urea containing Buffer A and analysed using a 0.8% agarose gel electrophoresis.

## Supplementary information

Supplementary information.
