# Supplementary material for: Tau protein- induced sequestration of the eukaryotic ribosome: Implications in neurodegenerative disease
Source: Sci Rep. 2020 Mar 23;10:5225. doi: 10.1038/s41598-020-61777-7 (PMC7090008; doi:10.1038/s41598-020-61777-7)
Supplement: Supplementary file 1 — Supplementary information. [file 41598_2020_61777_MOESM1_ESM.pdf]

Tau protein- induced sequestration of the eukaryotic ribosome: Implications in neurodegenerative disease

Senjuti Banerjee[1], Sehnaz Ferdosh[1], Amar Nath Ghosh[2] and Chandana Barat[1*]

[1]Department of Biotechnology, St. Xavier's College, Park Street, Kolkata-700016, West Bengal, India

[2]National Institute of Cholera and Enteric Diseases P-33, C.I.T. Road, Scheme XM, Beleghata, India

Running Title: ***Tau induced ribosome aggregation***

To whom correspondence should be addressed: Chandana Barat: Department of Biotechnology, St.Xavier's College (Autonomous), Under the University of Calcutta, Kolkata-700016; chandanasgb@yahoo.com; Tel.(033)2255-1101

Keywords: Tau protein, Ribosome, Aggregation, Alzheimer's Disease, Ribosomal RNA (rRNA), Intrinsically disordered protein

Supplementary figure 1

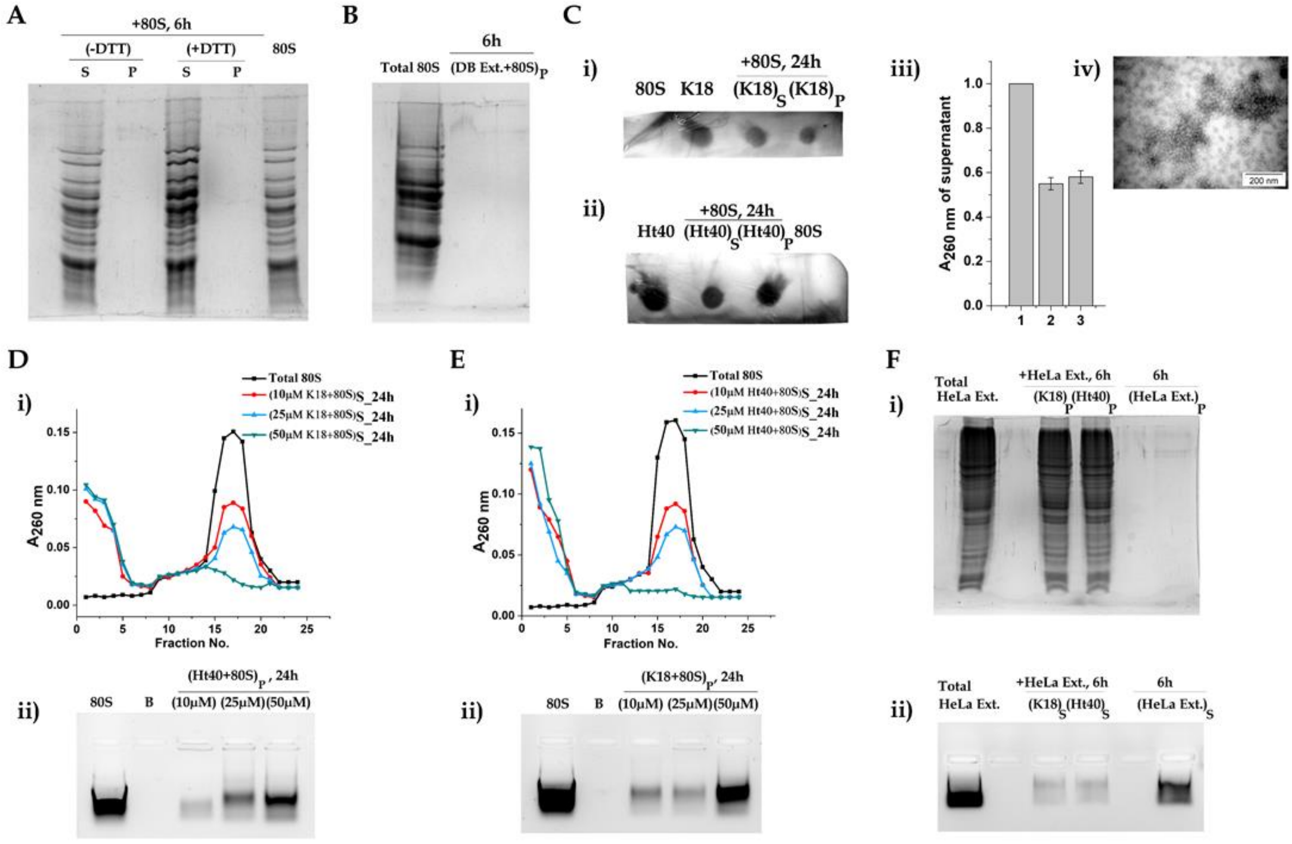

Tau-ribosome aggregation: tau partitioning and dependence on tau variant concentration

S1A. *SDS-PAGE analysis of supernatant and pellet fractions of ribosome alone and in presence of DTT*

0.1 µM of yeast 80S ribosome was incubated in buffer A in the presence and absence of 1 mM DTT for 6 hours at 37ºC. The reaction mixture was centrifuged and the pellet and TCA precipitated supernatant were analysed using a 12% SDS-PAGE. Lanes from left to right contain: (1) Supernatant of 80S alone, (2) Pellet of 80S alone, (3) Supernatant of 80S+1mM DTT, (4) Pellet of 80S+1mM DTT, (5) Total 80S ribosome used.

S1B. *Modification of ribosome physical integrity is specifically mediated by tau and not due to a bacterial constituent*

The *E.coli* BL21(DE3) cells were lysed using direct boiling method (used to purify K18, K19 and Ht40, as stated in materials and methods) and the lysate supernatant containing equivalent amount of protein ($A_{280\ nm}$) was incubated with 0.1 µM 80S for 6 hours at 37º C in Buffer A (25 mM Tris-HCl pH 7.5, 50 mM NaCl, 5 mM $MgCl_2$). The reaction mixture was centrifuged at 21,380 g, 4º C for 45 minutes. The pellet obtained was analysed using a 12% SDS-PAGE and stained using Coomassie Brilliant Blue. Lanes from left to right contain: (1) Total 80S and (2) Pellet of: Direct boiling (DB) extract + 80S.

S1C. *Analysis of supernatant and pellet fractions*

The supernatant and pellet obtained upon incubating 50 µM K18 or Ht40 with 0.1 µM purified yeast 80S ribosome for 24 hours were dotted on PVDF membrane and probed using monoclonal anti-K18 tau antibody and anti-Ht40 antibody (materials and methods)

i) Dots from left to right indicate: (1) 80S, (2) K18 Total Protein, (3) $(K18+80S)_{S\_24h}$, (4) $(K18+80S)_{P\_24h}$. The full-length dot-blot image is shown in Figure S4Ai.

ii) Dots from left to right indicate: (1) Ht40 Total Protein, (2) $(Ht40+80S)_{S\_24h}$, (3) $(Ht40+80S)_{P\_24h}$, (4) 80S. The full-length dot-blot image is shown in Figure S4Aii.

iii) Bar graphs showing relative amount of ribosomal RNA retained in the supernatant after 24 hours of incubating 50 µM K18 or Ht40 with 0.1 µM 80S ribosome.(1) $A_{260\ nm}$ of total 80S ribosome used (considered as 1 for calculations), (2) $A_{260\ nm}$ of $(K18+80S)_{S\_24h}$ and (3) $A_{260\ nm}$ of $(Ht40+80S)_{S\_24h}$

iv) Micrograph of supernatant fraction for K18-80S aggregation: 50 µM K18 was incubated with 0.1 µM 80S for 24 hours, centrifuged and the supernatant fraction was visualised using transmission electron microscopy.

S1D. *Effect of different concentrations of K18 on 0.1 µM yeast ribosome*

0.1µM 80S was incubated with different concentrations of K18 for 24 hours at 37°C and the outcome was analysed as described above.

i) Sedimentation profile of the supernatant fraction for K18-80S aggregation: Total 80S (■), $(10µM\ K18+80S)_{S\_24h}$ (●), $(25µM\ K18+80S)_{S\_24h}$ (▲) and $(50\ µM\ K18+80S)_{S\_24h}$ (▼);

ii) Agarose gel electrophoretic analysis of K18-80S aggregation pellet for ribosomal RNA; Lanes from left to right contain: (1) Total 80S, (2) Blank, (3) $(10µM\ K18+80S)_{P\_24h}$, (4) $(25µM\ K18+80S)_{P\_24h}$ and (5) $(50µM\ K18+80S)_{P\_24h}$;

S1E. *Effect of different concentrations of Ht40 on 0.1 µM yeast ribosome*

0.1 µM 80S was incubated with different concentrations of Ht40 for 24 hours at 37°C and the outcome was analysed as described above.

i) Sedimentation profile of the supernatant fraction for Ht40-80S aggregation: Total 80S (■), $(10µM\ Ht40+80S)_{S\_24h}$ (●), $(25µM\ Ht40+80S)_{S\_24h}$ (▲) and $(50\ µM\ Ht40+80S)_{S\_24h}$ (▼).

ii) Agarose gel electrophoretic analysis of Ht40-80S aggregation pellet for ribosomal RNA; Lanes from left to right contain: (1) Total 80S, (2) Blank, (3) $(10µM\ Ht40+80S)_{P\_24h}$, (4) $(25µM\ Ht40+80S)_{P\_24h}$ and (5) $(50µM\ Ht40+80S)_{P\_24h}$.

S1F. *Tau-HeLa extract aggregation*

The HeLa cell lysate or extract (ext) used in our experiments is a component of the human IVT kit. Equivalent $A_{260\ nm}$ units (to 0.1 µM of yeast 80S ribosome) of HeLa cell lysate was incubated with 50 µM K18 or Ht40 for 6 hours, under reducing conditions, at 37°C. The resultant reaction mixture was centrifuged at 21,380g, 4ºC for 45 minutes.

i) *SDS-PAGE analysis of Tau- HeLa Extract aggregation:* The pellets obtained were analysed using 12% SDS-PAGE. Lanes from left to right contain; (1) Total HeLa Extract used, (2) Blank, pellet of HeLa extract incubated with (3) K18 and (4) Ht40, (5) Blank, pellet of (6) HeLa extract incubated alone without tau for 6 hours.

ii*)* *Analysis of supernatant fraction obtained after 6 hours of Tau-HeLa ext aggregation for RNA*: The supernatant fractions were mixed with 4M urea solution such that the final urea concentration would be 1M and analysed using (0.8%) agarose gel electrophoresis. Lanes from left to right contain: (1) Total HeLa extract (ext) used, (2) Blank, Supernatant of: (3) HeLa Ext +K18 and (4) HeLa Ext +Ht40, (5) Blank, Supernatant of:(6) HeLa Ext alone incubated for 6 hours.

Supplementary figure 2

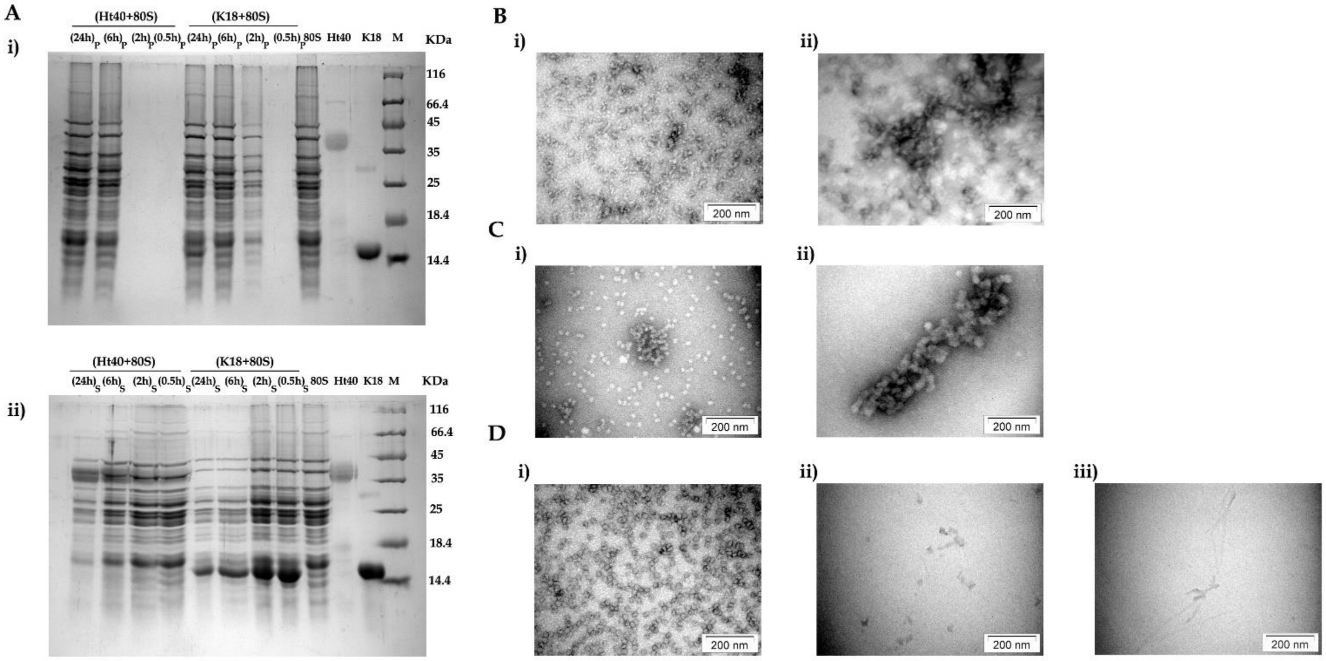

Time dependence and electron microscopy of tau induced yeast 80S ribosome aggregation

S2A. The yeast 80S ribosome (0.1 µM) was incubated with the tau variants for different time intervals, centrifuged and the TCA precipitated supernatant and pellet fractions were analysed using 12% SDS-PAGE.

i) *SDS-PAGE electrophoretic analysis of pellet obtained at different time intervals for K18-80S and Ht40-80S aggregation for the presence of ribosomal RNA*: Lanes from left to right contain; pellet of Ht40+80S aggregation: (1) (24h)$_P$, (2) (6h)$_P$, (3) (2h)$_P$, (4) (0.5h)$_P$ and pellet of K18+80S aggregation: (5) (24h)$_P$, (6) (6h)$_P$, (7) (2h)$_P$, (8) (0.5h)$_P$, (9) Total 80S ribosome, (10) Total Ht40, (11) Total K18, (12) Molecular weight marker

ii) *SDS-PAGE electrophoretic analysis of supernatant obtained at different time intervals for K18-80S and Ht40-80S aggregation for the presence of ribosomal RNA*: Lanes from left to right contain; supernatant of Ht40+80S aggregation: (1) (24h)$_S$, (2) (6h)$_S$, (3) (2h)$_S$, (4) (0.5h)$_S$ and supernatant of K18+80S aggregation: (5) (24h)$_S$, (6) (6h)$_S$, (7) (2h)$_S$, (8) (0.5h)$_S$, (9) Total 80S ribosome, (10) Total Ht40, (11) Total K18, (12) Molecular weight marker

Transmission electron microscopic analysis of aggregates: Micrographs were prepared from samples withdrawn at different time intervals (2 hours and 24 hours) from the initiation of incubation of 50µM of the tau protein variants; K18 (Bi-ii) and Ht40 (Ci-ii) in the presence of 0.1 µM of 80S ribosome. In the control micrographs K18 and Ht40 were incubated alone in the absence of ribosome under similar conditions for 24 hours.
S2B. Micrographs of K18 incubated with 80S for **i)** 2h shows loss of integrity of ribosomal particles and **iii)** 24h shows formation of large heterogeneous aggregates

S2C. Micrographs of Ht40 incubated with 80S for **i)**2h shows loss of integrity of ribosomal particles and **iii)** 24h shows formation of large heterogeneous aggregates. Figure S2D shows control micrographs of **i)** Total 80S ribosome and $(K18)_{\mathbf{24h}}$**(ii)** and $(Ht40)_{\mathbf{24h}}$ **(iii)** incubated for 24 hours in absence of the ribosome under the conditions used in our experiments (materials and methods).

Supplementary figure 3

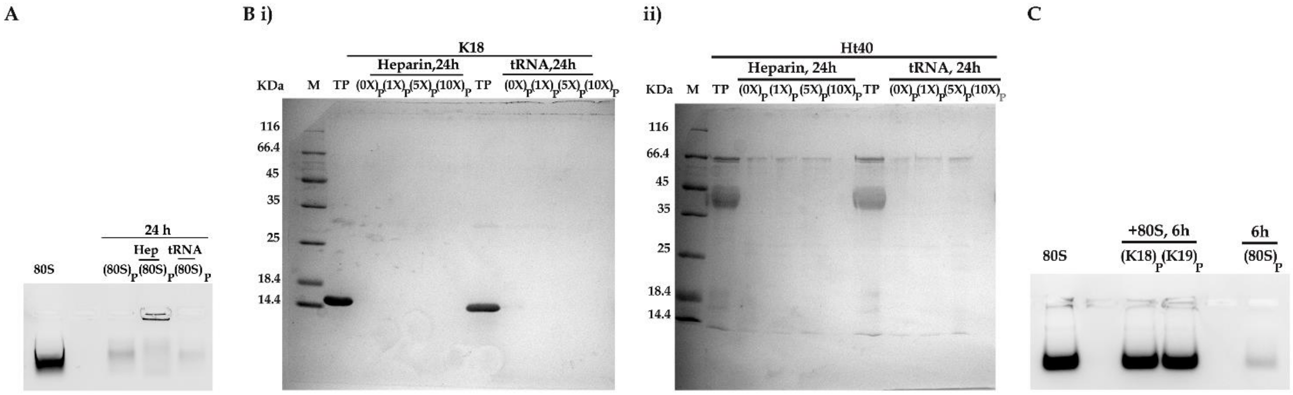

Heparin and tRNA do not increase the aggregation of 80S and tau variants and K19 tau variant can induce 80S ribosome aggregation

S3A. *Agarose gel electrophoretic analysis of insoluble pellet for ribosomal RNA*: 0.1 µM 80S was incubated in the absence and presence of 10x (x = 0.1 µM) heparin or tRNA, centrifuged and analysed using agarose gel electrophoresis. Lanes from left to right contain:(1) Total 80S, (2) $(80S)_{P\_24h}$, (3) $(80S+hep)_{P\_24h}$, (4) $(80S+tRNA)_{P\_24h}$.

S3B. *SDS-PAGE analysis of insoluble fraction for Tau*: 50 µM K18 or Ht40 was incubated with 0x, 1x, 5x and 10x (x=0.1 µM) of heparin and tRNA, centrifuged and analysed on SDS-PAGE.

i) Lanes from left to right contain: (1) Molecular weight marker (M), (2) K18 total protein, (3) $(K18+0x\ hep)_{P\_24h}$, (4) $(K18+1x\ hep)_{P\_24h}$, (5) $(K18+5x\ hep)_{P\_24h}$, (6) $(K18+10x\ hep)_{P\_24h}$, (7) K18 total protein, (8) $K18+0x\ tRNA)_{P\_24h}$, (9) $(K18+1x\ tRNA)_{P\_24h}$, (10) $(K18+5x\ tRNA)_{P\_24h}$, (11) $(K18+10x\ tRNA)_{P\_24h}$

ii) Lanes from left to right contain: (1) Molecular weight marker (M), (2) Ht40 total protein, (3) $(Ht40+0x\ hep)_{P\_24h}$, (4) $(Ht40+1x\ hep)_{P\_24h}$, (5) $(Ht40+5x\ hep)_{P\_24h}$, (6) $(Ht40+10x\ hep)_{P\_24h}$, (7) Ht40 total protein, (8) $(Ht40+0x\ tRNA)_{P\_24h}$, (9) $(Ht40+1x\ tRNA)_{P\_24h}$, (10) $(Ht40+5x\ tRNA)_{P\_24h}$, (11) $(Ht40+10x\ tRNA)_{P\_24h}$

S3C. *Comparison of the effect of K18 and K19 in inducing 80S aggregation:* 50 µM K18 or K19 were reduced in 1mM DTT in Buffer A (25 mM Tris HCl pH 7.5, 50 mM NaCl, 5 mM $MgCl_2$) at 37º C for 2 hours and incubated with 0.1 µM yeast 80S ribosome for 6 hours. The reaction mixture was centrifuged and the pellets were resuspended in 4 M urea and analysed using non-denaturing 0.8% agarose gel. Lanes from left to right contain: (1) Total 80S ribosome, (2) Blank, pellet of: (3) K18+80S and (4) K19+80S, (5) Blank, (6) pellet of 80S for 6 hours.

Supplementary figure 4

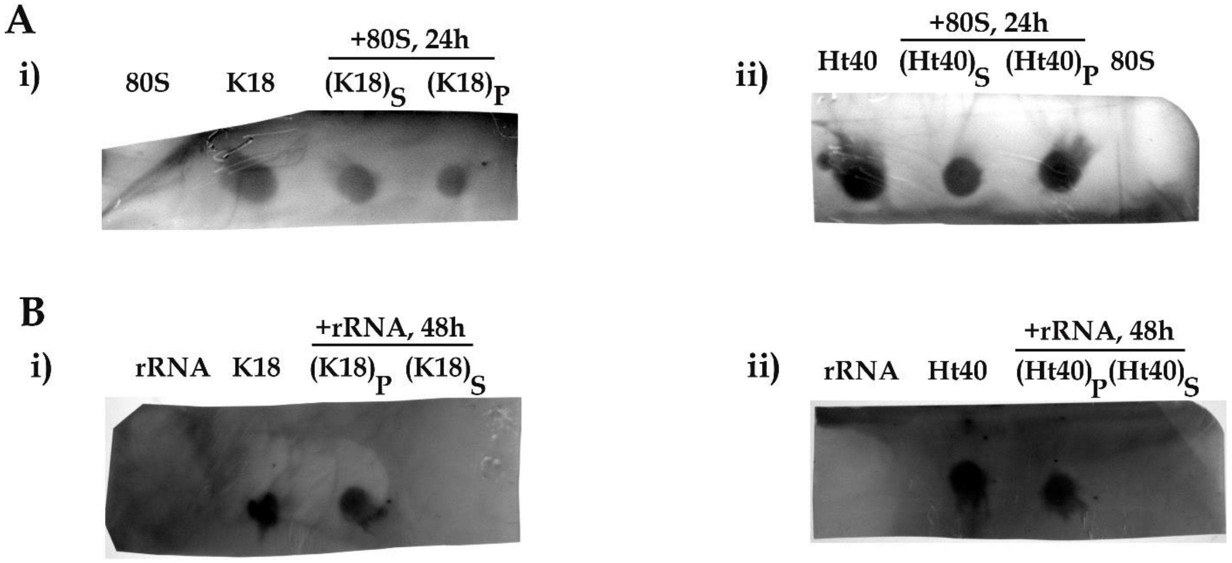

Original dot-blot images of Tau-80S aggregation and Tau-80SrRNA aggregation

S4A. A*nalysis of supernatant and pellet fractions obtained from Tau-80S aggregation*

The supernatant and pellet obtained upon incubating 50 µM K18 or Ht40 with 0.1 µM purified yeast 80S ribosome for 24 hours were dotted on PVDF membrane and probed using monoclonal anti-K18 tau antibody and anti-Ht40 antibody (materials and methods)

i) Dots from left to right indicate: (1) 80S, (2) K18 Total Protein, (3) (K18+80S)$_{S\_24h,}$ (4) (K18+80S)$_{P\_24h}$

ii) Dots from left to right indicate: (1) Ht40 Total Protein, (2) (Ht40+80S)$_{S\_24h,}$ (3) (Ht40+80S)$_{P\_24h}$, (4) 80S

S4B. *Analysis of supernatant and pellet obtained from Tau-80SrRNA aggregation*

50 µM K18 or Ht40 was incubated in the absence and in presence of 1 µM 80SrRNA for 48 hours at 37º C as described in “Materials and Methods”. The reaction mixture was centrifuged, the supernatant and pellet fractions were analysed using dot blot analysis with monoclonal anti-K18 tau antibody and anti-Ht40 antibody (materials and methods)

i) Dots from left to right indicate: Total 80SrRNA, Total K18, (K18+rRNA)$_{P\_48h}$, (K18+rRNA)$_{S\_48h}$

ii) Dots from left to right indicate: Total 80SrRNA, Total Ht40, (Ht40+rRNA)$_{P\_48h}$, (Ht40+rRNA)$_{S\_48h}$
